# The clinical value of repeat ultrasound-guided fine-needle aspiration biopsy in the management of Bethesda Category III thyroid nodules

**DOI:** 10.3389/fendo.2026.1750620

**Published:** 2026-02-27

**Authors:** Chong Chen, Qingfeng Fu, Rundong He, Yiming Guo, Shuai Zhang, Ping Sun, Le Zhou, Hui Sun

**Affiliations:** Department of Thyroid Surgery, China-Japan Union Hospital of Jilin University, Jilin Provincial Key Laboratory of Translational Medicine in Surgery, Jilin Provincial Engineering, Laboratory of Thyroid Disease Prevention and Treatment, Changchun, China

**Keywords:** ACR TI-RADS, bethesda Category III, fine-needle aspiration cytology, repeat fine-needle aspiration biopsy, Thyroid nodule

## Abstract

**Objective:**

This study aimed to evaluate the clinical value of repeat fine-needle aspiration biopsy (rFNAB) for thyroid nodules initially classified as Bethesda Category III, to explore the optimal timing for repeat biopsy, and to optimize the biopsy strategy by integrating ultrasonographic characteristics.

**Methods:**

We retrospectively analyzed the clinical data of 109 patients (114 nodules) who underwent rFNAB at our hospital from December 2020 to December 2025, including 87 females and 22 males. Based on rFNAB results, the nodules were divided into a definitive diagnosis group (93 nodules) and a non-definitive diagnosis group (21 nodules). The definitive diagnosis group was further subdivided into a malignant group (62 nodules, Bethesda V/VI) and a benign group (31 nodules, Bethesda II). We recorded biopsy results, intervals between biopsies, postoperative pathological findings, and ultrasonographic features. Statistical differences between groups were analyzed. Statistical methods included the χ² test, Fisher’s exact test, binary logistic regression analysis, and ROC curve analysis.

**Results:**

Among the 114 Bethesda Category III nodules, 93 (81.6%) obtained a definitive diagnosis through rFNAB, including 62 malignant (66.7%) and 31 benign (33.3%) nodules; 21 nodules (18.4%) remained non-diagnostic. Forty-seven patients with malignant rFNAB results underwent surgical treatment, including 9 (19.1%) in the <3-month interval group and 38 (80.9%) in the ≥3-month interval group. No significant differences were observed in lymph node metastasis rates or recurrence risk stratification between the two groups. Among the ultrasonographic characteristics of the malignant, benign, and non-diagnostic groups, only calcification type showed a statistically significant difference (P < 0.05). Additionally, ROC curve analysis confirmed the diagnostic efficacy of the TI-RADS score for Bethesda Category III nodules (AUC = 0.746). The maximum Youden index (0.403) was achieved at a cut-off value of 8 points (specificity 83.9%, sensitivity 56.5%).

**Conclusion:**

Repeat fine-needle aspiration biopsy (rFNAB) significantly improves the diagnostic rate for thyroid nodules initially diagnosed as Bethesda Category III, and the time interval between the two biopsies does not affect diagnostic performance. For the follow-up of nodules with an initial Bethesda Category III FNAB result, rFNAB is recommended when the TI-RADS score is ≥8, especially if suspicious ultrasonographic signs such as new or persistent microcalcifications are present. During the procedure, multi-point sampling should target non-calcified areas at the nodule periphery to improve the accuracy and reliability of rFNAB.

## 1 Introduction

In recent years, the incidence of thyroid nodules has been increasing annually. In the general population, the detection rate of thyroid nodules can reach 60%, of which approximately 5% are ultimately confirmed to be malignant (1). Therefore, determining the nature of thyroid nodules is of paramount importance. Fine-needle aspiration biopsy (FNAB) is the preferred method for diagnosing thyroid nodules, with studies reporting a sensitivity of up to 82% and specificity approaching 100% (2, 3).However, initial FNAB results may be non-diagnostic or indeterminate due to insufficient sampling or ambiguous cytological features, leading to non-diagnostic or uncertain results in up to 20%-30% of cases (4). Repeated fine-needle aspiration biopsy (rFNAB) can serve as an effective supplementary method. Studies have shown that rFNAB can significantly improve the diagnostic rate and reduce unnecessary surgeries. Nevertheless, the clinical value and optimal timing of rFNAB remain subjects of debate. This study aims to evaluate the impact of rFNAB on the clinical management of Bethesda Category III nodules and to further optimize the biopsy strategy.

## 2 Materials and methods

### 2.1 Study population

This study retrospectively analyzed the clinical data of 109 patients (114 nodules) who underwent rFNAB at the Department of Thyroid Surgery, China-Japan Union Hospital of Jilin University from December 2020 to January 2025, including 87 females and 22 males. Based on rFNAB results, the nodules were divided into a definitive diagnosis group (93 nodules) and a non-definitive diagnosis group (21 nodules). The definitive diagnosis group was further subdivided into a malignant group (62 nodules, Bethesda V/VI) and a benign group (31 nodules, Bethesda II), while the non-definitive diagnosis group included Bethesda I/III/IV nodules.

### 2.2 Inclusion and exclusion criteria

Inclusion criteria were: ① initial FNAB result of Bethesda Category III (atypia of undetermined significance or follicular lesion of undetermined significance); ② underwent two rFNAB procedures at our center with complete ultrasonographic features and pathological data. Exclusion criteria were: ① lack of complete pathological data; ② received other treatments during follow-up; ③ the two biopsies targeted different nodules. All patients and their families provided informed consent.

### 2.3 Research methods

#### 2.3.1 Thyroid biopsy procedure

The puncture site and surrounding skin (≥5 cm) were routinely disinfected preoperatively. The ultrasound probe was sterilized using a sterile sheath isolation technique. After identifying the nodule’s location, size, margins, calcifications, and blood supply using high-resolution real-time ultrasound, a 22–23 G fine needle was inserted into the nodule under ultrasound guidance. The needle was moved back and forth 10–20 times, with this process repeated three times per nodule. The core material was expelled onto glass slides, fixed with 95% ethanol, and immediately sent for cytopathological examination.

#### 2.3.2 Data collection

Cytological specimens were classified by pathologists according to the Bethesda system, with diagnostic categories as follows: Category I (non-diagnostic), Category II (benign), Category III (atypia of undetermined significance or follicular lesion of undetermined significance), Category IV (follicular neoplasm or suspicious for follicular neoplasm), Category V (suspicious for malignancy), and Category VI (malignant). Demographic data and clinical characteristics of the nodules were recorded, including maximum diameter, echogenicity (hypoechoic/other), aspect ratio, shape (regular/irregular), calcification type (microcalcification/other), and TI-RADS classification. For patients with malignant rFNAB results who underwent surgery, surgical pathological results were recorded, including lesion size, lymph node metastasis status, and recurrence risk stratification (low, intermediate, high risk).

#### 2.3.3 Statistical analysis

Statistical analysis was performed using SPSS 25.0 software. Categorical data are presented as proportions (%), and group comparisons were conducted using the chi-square test, Fisher’s exact test, or one-way ANOVA. Binary logistic regression analysis and ROC curve analysis were also employed. A P-value < 0.05 was considered statistically significant.

## 3 Results

### 3.1 Diagnostic performance of repeat FNAB

Among the 114 Bethesda Category III nodules, 93 (81.6%) obtained a definitive diagnosis through rFNAB, including 62 malignant (66.7%) and 31 benign (33.3%) nodules. Twenty-one nodules (18.4%) remained non-diagnostic (**Table 1**).

**Table 1:** rFNAB results of thyroid bethesda category III nodules.

Diagnostic group	Number (%)	rFNAB result	Number (%)
Diagnostic Group Number (%)	21(18.4)	Category I	1(0.9)
		Category III	20(17.5)
		Category IV	0
Definitive Group	93(81.6)	Category II	31(27.2)
		Category V	10(8.8)
		Category VI	52(45.6)

### 3.2 Postoperative pathological analysis

Forty-seven patients with malignant rFNAB results underwent surgical treatment. Their clinical characteristics were as follows:

There were 9 patients (20.8%) with an rFNAB interval of <3 months. Among them, 3 had lesion diameters ≥1 cm, 2 had lymph node metastasis, and postoperative recurrence risk stratification showed 9 (100.0%) as low risk. There were 38 patients (79.2%) with an rFNAB interval of ≥3 months. Among them, 4 had lesion diameters ≥1 cm, 8 had lymph node metastasis, and postoperative recurrence risk stratification showed 34 (89.5%) as low risk and 4 (10.5%) as intermediate risk. The lymph node metastasis rates were similar between the two groups (22.2% in the <3-month group vs. 21.1% in the ≥3-month group) (**Table 2**).

**Table 2:** Postoperative pathological findings in patients with malignant rFNAB thyroid nodules [n (%)].

Interval	Surgery cases	Lesion max diameter (cm)		Lymph node metastasis (n)		Recurrence risk stratification	
		<1	≥1	Yes	No	Low risk	Intermediate risk
<3 months	9	6(66.7)	3(33.3)	2(22.2)	7(77.8)	9(100.0)	
≥3months	38	34(89.5)	4(10.5)	8(21.0)	30(79.0)	34(89.5)	4(10.5)

### 3.3 Analysis of ultrasonographic features and biopsy intervals in bethesda category III nodules

Among the 114 nodules initially diagnosed as Bethesda Category III, comparisons among the malignant, non-diagnostic, and benign groups regarding maximum diameter, internal echogenicity, aspect ratio, shape, TI-RADS classification, and interval between the two biopsies showed no statistically significant differences (P > 0.05). However, further pairwise comparisons revealed that the differences in calcification type between the malignant group and the benign group, and between the malignant group and the non-diagnostic group, were statistically significant (P < 0.05), suggesting that calcification characteristics may have reference value for distinguishing benign from malignant Bethesda Category III nodules (**Table 3**).

**Table 3:** Characteristics of ultrasound features in patients with thyroid nodules.

Group	Number of nodules	Time interval		Maximum diameter		Echogenicity		Aspect ratio	
		<3 months	≥3 months	<1cm	≥1cm	Hypoechoic	Other	<1	≥1
Malignant	62	9	53	47	15	59	3	30	32
Benign	31	3	28	26	5	29	2	21	10
Non-Definitive Diagnosis	21	1	20	17	3	20	1	15	6
X²		–		1.259		–		5.11	
P		0.528		0.533		1.000		0.078	
Group	Number of Nodules	Shape		Calcification		TI-RADS			
	枚	Regular	Irregular	Microcalcification	Other	4	5		
Malignant	62	47	15	32	30	22	40		
Benign	31	27	4	3	28	17	14		
Non-Definitive Diagnosis	21	13	8	3	18	10	11		
X²		4.415		20.558		3.385			
P		0.11		0.001		0.184			

### 3.4 Diagnostic efficacy analysis of TI-RADS scoring

**Figure 1:**
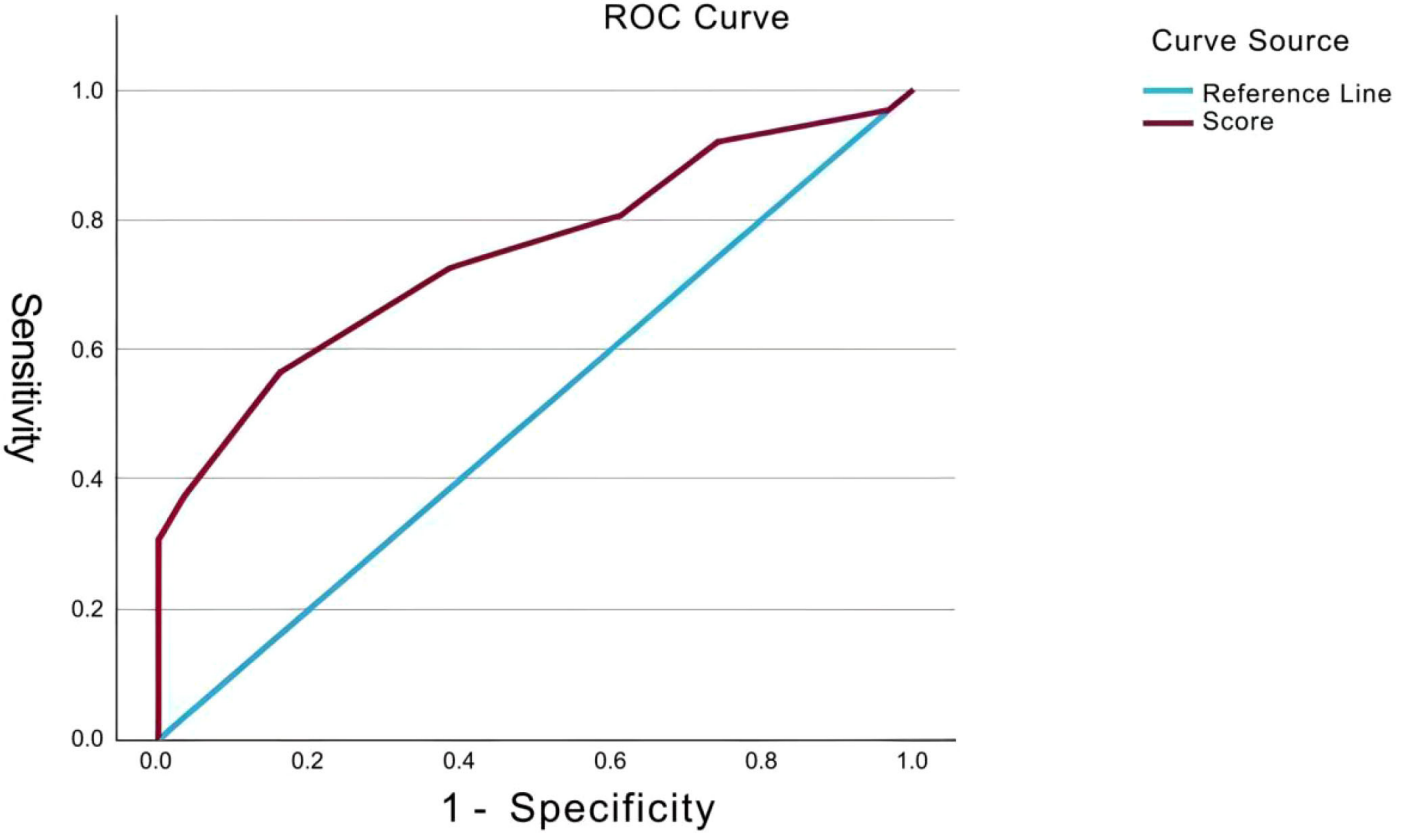
Receiver operating characteristic (ROC) curve of TI-RADS score for differentiating benign and malignant thyroid nodules by rFNAB.

Using rFNAB cytopathological results as the gold standard, the receiver operating characteristic (ROC) curve of TI-RADS scoring for differentiating benign from malignant among initially biopsied Bethesda Category III nodules is shown in **Figure 1**. The area under the curve (AUC) was 0.746 (95% CI: [0.648–0.844], P < 0.001), indicating good overall diagnostic value of this scoring system. As shown in **Table 4**, the statistical optimal cut-off value corresponding to the maximum Youden index (0.403) was 7.5 points, with a sensitivity of 56.5% and specificity of 83.9%. Notably, a cut-off value of 6.5 also demonstrated good diagnostic performance, with a sensitivity of 72.6% and specificity of 61.3%, achieving a better clinical utility balance (**Table 4**).

**Table 4:** Efficacy Analysis of TI-RADS Scoring in Diagnosing Benign and Malignant rFNAB Thyroid Nodules.

Score	Sensitivity	Specificity	Youden Index
≥ 2.0	1.000	0.000	.000
≥ 3.5	.968	.032	.000
≥ 4.5	.919	.258	.177
≥ 5.5	.806	.387	.194
**≥ 6.5**	**.726**	**.613**	**.339**
**≥ 7.5**	**.565**	**.839**	**.403**
≥ 8.5	.371	.968	.339
≥ 9.5	.306	1.000	.306
≥ 10.5	.177	1.000	.177
≥ 11.5	.161	1.000	.161
≥ 12.5	.032	1.000	.032
≥ 14.0	.000	1.000	.000

The maximum Youden Index is 0.403, corresponding to a cut-off value of ≥7.5. Bolded rows indicate key cut-off values with significant clinical reference importance.

## 4 Discussion

Thyroid nodules are a common clinical endocrine disorder, and their risk of malignant transformation has always been a focus of clinical attention. Fine-needle aspiration biopsy (FNAB), as the gold standard for preoperative diagnosis of thyroid nodules, has been widely used. However, the incidence of Bethesda Category III (AUS/FLUS) is approximately 10%–20% among FNAB results, making AUS/FLUS a relatively common diagnostic category and posing significant challenges for the diagnosis and clinical management of thyroid nodules. The latest edition of The Bethesda System for Reporting Thyroid Cytopathology (TBSRTC, 2023) indicates that the average risk of malignancy for Bethesda Category III (AUS/FLUS) is as high as 22% (range 13%–30%) (4). This risk level is significantly higher than that of benign nodules but insufficient to warrant immediate surgical excision. Therefore, clinicians face a dilemma in managing these nodules—they cannot ignore the potential malignancy, nor do they want to lead to unnecessary surgeries due to over-diagnosis. TBSRTC recommends repeat fine-needle aspiration biopsy (rFNAB) for Bethesda Category III (AUS/FLUS) nodules to further clarify their nature (4). However, there are currently no clear guidelines regarding the timing of rFNAB, its diagnostic value, or which nodules should preferentially undergo rFNAB. These issues have become hot topics in research and urgently require further investigation and clarification to optimize the clinical management strategy for thyroid nodules.

### 4.1 Value of repeat FNAB in diagnosing Bethesda Category III (AUS/FLUS) thyroid nodules

To further clarify the nature of Bethesda Category III (AUS/FLUS) thyroid nodules, The Bethesda System for Reporting Thyroid Cytopathology (TBSRTC) recommends performing repeat fine-needle aspiration biopsy (rFNAB) under ultrasound guidance (4). In this study, for nodules with an initial FNAB result of Bethesda Category III, 81.6% obtained a definitive diagnosis after rFNAB, with a malignancy rate of 54.4% and a benign rate of 27.2%. This result significantly improved the reclassification rate of nodules and substantially increased the detection rate of malignant nodules. Numerous studies have confirmed the significant value of rFNAB in improving diagnostic accuracy. A systematic review and meta-analysis including 3932 AUS/FLUS thyroid nodules showed that rFNAB reclassified approximately 70% of AUS/FLUS samples into more definitive cytological categories, with a benign detection rate close to 50% (5), thereby avoiding unnecessary surgery for more patients. However, in international studies, the detection rate of benign nodules is higher than that of malignant nodules. This finding is inconsistent with the results of this study and studies by YU Lulu, ZHAO Na, et al. (6–8). This discrepancy may be related to the domestic tendency to select nodules with high-risk features for repeat biopsy, such as nodule growth or ultrasound suggesting malignant features. Additionally, a more proactive attitude towards interpreting repeat biopsy pathological results domestically might also contribute to the higher proportion of malignant results, thus improving the detection rate of malignant tumors. Nevertheless, these results indicate that rFNAB can, to some extent, compensate for the limitations of initial FNAB and provide a more reliable basis for clinical decision-making.

### 4.2 Timing of repeat US-FNAB and its impact on patients

In the management of thyroid nodules, the timing of repeat FNAB (rFNAB) after an initial Bethesda Category III (AUS/FLUS) result is an important issue in clinical decision-making. The Bethesda guidelines recommend waiting 3 months after the initial biopsy for the second biopsy (4). Some previous studies support this view, advising against repeating FNAB (rFNAB) on the same nodule within a short period to avoid misinterpretation of cellular atypia caused by the initial FNAB as cytological features of thyroid cancer (4, 9). However, in recent years, more studies have suggested that the time interval between two FNABs does not affect diagnostic performance, and repeating FNAB within a short time does not increase false-positive results (10–12) In this study, no significant difference was found between the definitive diagnosis group and the non-definitive diagnosis group regarding whether rFNAB was performed before or after 3 months, which is consistent with the views of most researchers. Some studies suggest that rFNAB should be performed within a short period for Bethesda Category III nodules to avoid poor patient prognosis (13). However, in this study, we analyzed the postoperative pathology of patients who underwent surgery after a follow-up period exceeding 3 months and found that only 10.5% of patients had an intermediate recurrence risk, while the rest were low risk, with no serious consequences due to delayed treatment. KOH et al.’s study also found that in the absence of lateral cervical lymph node metastasis on ultrasound, delayed surgery had no significant effect on extra thyroidal extension and the extent of lymph node metastasis (14). Furthermore, studies have suggested that papillary thyroid carcinomas (PTC) smaller than 1.5 cm progress relatively slowly, have a low rate of lymph node metastasis, and delayed surgery has no adverse effect on patient prognosis in the absence of other high-risk factors (14, 15). Therefore, patients with an initial FNAB result of Bethesda Category III and no high-risk factors can undergo long-term follow-up with ultrasound to monitor feature changes and decide on further clinical management strategies. However, the author believes that by combining the ultrasonographic features of the nodule, repeat biopsy can be performed at a more appropriate time. Monitoring changes in the nodule’s ultrasonographic features (especially the appearance of microcalcifications) and the TI-RADS score (especially ≥8) can improve diagnostic accuracy while reducing unnecessary biopsies, avoiding over-medicalization.

### 4.3 Ultrasonographic features of nodules undergoing repeat US-FNAB

This study found that the presence of microcalcifications in the benign, malignant, and non-diagnostic groups was of great significance in predicting the nature of Bethesda Category III thyroid nodules. The research results of Abdulrahman S Alamri, Yoo, W. S., et al. also proved that highly suspicious ultrasonographic features of microcalcifications aid in the diagnosis of thyroid cancer among Bethesda Category III (AUS/FLUS) nodules (16, 17). Therefore, during follow-up, heightened vigilance should be maintained for nodules accompanied by microcalcifications, and further rFNAB examination should be performed to clarify the diagnosis. However, in the author’s study, hypoechogenicity showed no statistical difference between the malignant group and the benign group, or between the malignant group and the non-diagnostic group, which is inconsistent with the study by Abdulrahman S Alamri et al. This may be related to the fact that physicians in this study initially selected nodules with an aspect ratio greater than 1, irregular shape, and hypoechogenicity for FNAB.

### 4.4 Diagnostic efficacy analysis of TI-RADS scoring for nodules undergoing repeat US-FNAB

This study confirmed through ROC analysis that TI-RADS scoring is an effective tool for differentiating benign from malignant among initially biopsied Bethesda Category III nodules (AUC = 0.746), which is consistent with conclusions from previous relevant studies (18, 19). The ROC curve coordinates showed that the maximum Youden index (0.403) occurred at a cut-off value of 7.5, with a specificity of 83.9% and sensitivity of 56.5%. Given that scores in clinical practice are integers, and based on the principles of ROC curve construction, this cut-off value is statistically entirely equivalent to the clinical decision rule “TI-RADS score ≥ 8 points”. Therefore, from a statistical perspective, this study recommends using ≥8 points as the clinical operational threshold for performing biopsy. However, when applying this threshold, one must be cautious of its relatively low sensitivity; for nodules with scores close to this threshold, comprehensive judgment combining ultrasonographic features is advisable. It is worth noting that a cut-off value of 6.5 (corresponding to a “score ≥ 7 points”) also demonstrated good diagnostic performance, with a sensitivity (72.6%) and specificity (61.3%) achieving a better balance for clinical utility. How to choose the operational cut-off value is essentially a clinical decision problem, depending on our assessment of the relative harms of two types of errors: “false negatives” (missed diagnosis) and “false positives” (over-biopsy). The accurate selection of the final threshold should be based on a specific weighing of the clinical situation.

### 4.5 Optimization of FNAB

This study thoroughly investigated potential factors influencing Bethesda Category III results in thyroid nodule fine-needle aspiration biopsy (FNAB). Statistical analysis of age and gender among the benign, malignant, and non-diagnostic groups showed that these factors were not statistically significant, which is inconsistent with previous study results (20). The author believes this result may be related to the following factors: Firstly, the relatively small sample size of this study may not have sufficiently revealed differences between groups; Secondly, physicians in this study tended to select nodules with higher TI-RADS classifications for repeat FNAB (rFNAB), which may have led to a relatively concentrated age and gender distribution in the population with initial Bethesda Category III results. Currently, the unsatisfactory rate of FNA specimens is as high as 20% (21). Calcifications, cystic changes, and necrotic areas within the nodule can affect the quality of sampling, while nodule size and an aspect ratio greater than 1 also influence the satisfaction level of the sampling (22–24). Additionally, the operator’s skill level, the adequacy of specimen preparation, and the typicality of cytological features of malignancy can also affect the pathologist’s judgment. To improve the specimen quality and diagnostic accuracy of FNAB, the biopsy strategy should be optimized based on the ultrasonographic features of the nodule. For large solid nodules, priority should be given to sampling the peripheral cell-rich areas, avoiding central necrosis or liquefaction areas; for cystic-solid nodules, the solid components need to be precisely targeted, and cystic fluid should be aspirated for examination. When biopsying very small nodules with a diameter less than 3 mm, the first needle must ensure precise targeting under ultrasound guidance to avoid bleeding that obscures the target. Furthermore, the needle insertion strategy should be adjusted according to the calcification morphology: peripheral calcifications can be targeted at the central area, while coarse or arc-shaped calcifications should be sampled from their edges or internal solid parts. The aforementioned targeted measures can effectively improve the success rate of sampling and the reliability of pathological evaluation, reducing diagnostic uncertainty caused by improper sampling (25, 26). The author re-reviewed the data: there were 21 nodules with Bethesda Category III results on both biopsies, among which 5 patients underwent surgery and postoperative pathology reported malignancy. Relevant studies also suggest that malignancy rates are higher for nodules with non-diagnostic results after more than two biopsies. Surgical treatment is recommended, which is consistent with the author’s view (26). Additionally, molecular testing can guide surgical decision-making in cases of insufficient cytological evidence. Among the 21 nodules with Bethesda Category III results on both biopsies in this study, 3 patients underwent molecular testing, all with positive results; postoperative pathology confirmed malignancy in 2 cases, and 1 received radiofrequency ablation therapy. In a 2015 survey of clinical practice patterns in the United States, 38.8% of 820 respondents performed molecular testing after an initial AUS/FLUS result (27). A high negative predictive value of 95% became the basis for physicians to postpone surgery (28). Another study also pointed out that molecular testing is more cost-effective than diagnostic lobectomy for Bethesda Category III nodules (29). Therefore, combining molecular testing technology can serve as a supplement to rFNAB, providing stronger guidance for surgical decision-making.

### 4.6 Limitations and future perspectives

This study possesses certain inherent limitations that warrant careful consideration. As a retrospective analysis conducted at a single institution, our findings may be influenced by selection bias and institutional-specific clinical practices. Although stringent inclusion and exclusion criteria were applied to enhance data reliability, the relatively limited sample size may restrict the generalizability of our conclusions. Furthermore, the observed higher malignancy rate in our cohort could partially be attributed to the clinical tendency to prioritize repeat biopsy for nodules exhibiting suspicious ultrasonographic features, such as microcalcifications or high TI-RADS scores. Future research should aim to validate these findings through prospective, multicenter studies with larger and more diverse patient populations. Additionally, integrating molecular testing with repeated cytological evaluation may further refine risk stratification and guide personalized management strategies for Bethesda Category III nodules.

## 5 Conclusion

Bethesda Category III thyroid nodules carry a relatively high risk of malignancy. Repeat fine-needle aspiration biopsy (rFNAB) significantly improves the diagnostic rate for thyroid nodules initially diagnosed as Bethesda Category III,and our study showed the time interval between the two biopsies may not affect diagnostic performance. For the follow-up of nodules with an initial Bethesda Category III FNAB result, rFNAB is recommended when the TI-RADS score is ≥8, especially if suspicious ultrasonographic signs such as new or persistent microcalcifications are present. During the procedure, multi-point sampling should target non-calcified areas at the nodule periphery to improve the accuracy and reliability of rFNAB. On this basis, future combination with molecular marker testing is expected to further optimize the precise management of such nodules, ultimately advancing their diagnosis and treatment strategies towards individuation and refinement.

## Data Availability

The raw data supporting the conclusions of this article will be made available by the authors, without undue reservation.
